# The dynamics of shapes of vesicle membranes with time dependent spontaneous curvature

**DOI:** 10.1371/journal.pone.0227562

**Published:** 2020-01-14

**Authors:** R. A. Barrio, Tomas Alarcon, A. Hernandez-Machado

**Affiliations:** 1 Instituto de Física, U.N.A.M., Apartado Postal 20-364, 01000 Mexico D.F., Mexico; 2 ICREA, Pg. Lluís Companys 23, 08010 Barcelona, Spain; 3 Centre de Recerca Matemàtica, Edifici C, Campus de Bellaterra, 08193 Bellaterra (Barcelona), Spain; 4 Departament de Matemàtiques, Universitat Autònoma de Barcelona, 08193 Bellaterra (Barcelona), Spain; 5 Barcelona Graduate School of Mathematics (BGSMath), Barcelona, Spain; 6 Departament de Física de la Matèria Condensada, Facultat de Física, Universitat de Barcelona, Diagonal 645, E-08028 Barcelona, Spain; 7 Institute of Nanoscience and Nanotechnology (IN2UB), Universitat de Barcelona, Barcelona, Spain; Peking University, CHINA

## Abstract

We study the time evolution of the shape of a vesicle membrane under time-dependent spontaneous curvature by means of phase-field model. We introduce the variation in time of the spontaneous curvature via a second field which represents the concentration of a substance that anchors with the lipid bilayer thus changing the local curvature and producing constriction. This constriction is mediated by the action on the membrane of an structure resembling the role of a Z ring. Our phase-field model is able to reproduce a number of different shapes that have been experimentally observed. Different shapes are associated with different constraints imposed upon the model regarding conservation of membrane area. In particular, we show that if area is conserved our model reproduces the so-called L-form shape. By contrast, if the area of the membrane is allowed to grow, our model reproduces the formation of a septum in the vicinity of the constriction. Furthermore, we propose a new term in the free energy which allows the membrane to evolve towards eventual pinching.

## Introduction

A commonly used experimental setup to study the fundamental elements involved in constriction of a lipid bilayer *in vitro* consists of using a mixture of lipids and FtsZ protein. The lipids organise themselves into rodlike liposomes which resemble the structure of the bacterium membrane. The FtsZ protein, in turn, accumulates on this rod-shaped structure forming rings scattered over the length of the liposome. The results of these experiments demonstrate that FtsZ is enough for assembling Z-rings and therefore to generate constriction forces on the liposome [1–3].

The early stages of the constrictive process is well described by the aforementioned experiments. In *in vitro* experiments, such as the ones performed in [1–3], the system evolves under area conservation constraints and the shape of the membrane evolves towards the so-called L forms (which are typically pear-shaped membranes) [4]. These results have motivated a number of modelling and theoretical studies examining different aspects of the early stages of constriction associated with linear deformations [5–16]. The long-time behaviour has been much less studied since it exhibits remarkable variability depending on the exact conditions under which the experiment is carried out.

To address the long-time behaviour, we propose a model capable of anlysing the system in the non-linear (long-time) regime corresponding to large deformations of the fluid membrane. The proposed mechanism consists of assuming that local spontaneous curvature can be dynamically changed by means of a number of proteins that inserts within the lipid bilayer and act as wedges in the vesicle membrane [17]. In particular, we propose a phase-field model which accounts for the bending force exerted on the membrane that has been previously used to study the early stages of FtsZ-induced constriction in liposomes [18]. Whilst the basic bending energy model under appropriate volume-area constraints produces rod-shaped patterns, the presence of the protein is necessary to produce constriction. This model is now extended in two ways. First, the model itself is modified to account for a new energy term which leads to eventual pinching. The second extension consists of studying this new model under conditions where the area of the membrane is allowed to grow. These two additions allow us to produce an enriched sample of possible shapes. Specifically, Picallo et al. [18] proved the existence of a short-time dynamical instability which provides a sufficient condition for constrain to occur, thus allowing us to control the process of ring formation. In this paper, using a non-linear, non-local phase-field model, we generalise our previous model to describe the dynamics of the membrane up to pinching. A novel contribution to the model is a new term that induces pinching at the end of the division process. Regarding previous phase-field modelling results in biomembranes, references [19, 20] propose a phase-field formulation of a bending energy model. The effect of constant spontaneous curvature was explored in reference [21]. In [8], a linearised model accounting for the effects of the coupling between a membrane and the FtsZ protein was proposed. Liu and Guo [22] have formulated a phase-field model where the FtsZ ring is assumed to be in quasi-steady state, thus neglecting the initial stage where the ring is formed. They consider that the effect of the ring on the membrane is via deformation obeying linear elasticity. It is important to note that all of the aforementioned models lead to a well-defined stationary state for constricted shapes. We obtain, under the usual constraint of area conservation, the L-form pattern. Moreover, when such condition is relaxed and we allow for the area of the membrane to grow (as we allow the surface tension to be small relative to the bending energy), other patterns are observed. In particular, our model produces a septum-like shape.

Our model is based on a free energy whose terms account for different physical processes occurring during the evolution of the shape of the membrane. The effect of the protein on membrane constriction is accounted for by the bending energy term. Full constriction is described by means of a new energy term. This energy term does not directly account for the effects of the curvature-inducing protein but for the effect of other proteins acting after constriction has occurred and responsible for actual pinching.

Physically, the rationale for the form of the pinching energy contribution is that the proximity of the membrane elements at the bottleneck region is energetically costly and thus the membrane tends to pinch. We show that the process leading to pinching consists of three temporal regimes. The short-time behaviour is determined by an instability induced by the spontaneous curvature. If instability occurs, this regime is followed by a non-linear process where rings of constriction are formed by accumulation of protein (i.e. high spontaneous curvature) on localised regions on the membrane. Last, a long-time regime ensues where energy contributions associated to pinching take over. We show that the long-term dynamics of the membrane shape is critically affected by the constraints regarding area conservation imposed upon the system. A feature of our model is that the system does not reach an equilibrium state with constant curvature, as it is the case in linear models. This property is essential to the evolution of the system until the system is close to pinching, as it allows the system to constrain until such regime is reached.

## The model

We assume that the non-linear dynamics of the system is dominated by a bending energy functional, following the well-known approach of Canham-Helfrich [23]. Our model is of the Ginzburg-Landau type, where the dynamics is described by the relaxation to a minimum of a free energy functional. From the hydrodynamical point of view our model corresponds to the limit of very small Reynolds numbers where the surrounding fluid is slaved to the dynamics of the membrane. Our model for the dynamics of the membrane has been rigorously derived, by means of an asymptotic expansion, in references [19, 20]. The model accounting for the effects of the protein has been explained and analysed in detail, including a linear stability analysis, in [18]. Specifically, we implement a Canham-Helfrich minimization scheme by means of a phase field model for the order parameter *ϕ*, that takes the value *ϕ* = 1 inside the vesicle and *ϕ* = −1 out of the vesicle. Hence, the level set *ϕ* = 0 gives us the membrane location. We further consider a second field *u*, which represents the local concentration of FtsZ protein in the domain. Both the protein concentration, *u*, and the order parameter, *ϕ*, are conserved quantities and hence their dynamics is given by:

$$\frac{\partial \phi }{\partial t}=D_{\phi }\nabla^{2}(\frac{\delta F}{\delta \phi })$$ (1)

$$\frac{\partial u}{\partial t}=D_{u}\nabla^{2}(\frac{\delta F}{\delta u})$$ (2)

where *D*_*ϕ*_ and *D*_*u*_ are the corresponding diffusion coefficients, providing the time scales for the system. As a consequence of Eqs (1) and (2), both *ϕ* and *u* are conserved quantities. The total free energy of the vesicle-protein system, *F*, is given by:

$$\begin{array}{c} F=\int_{V}(A_{b}\Phi_{b}^{2}+A_{s}V_{s}+A_{f}V_{f}+A_{p}V_{p}+{\sigma |\nabla \phi |}^{2})dV \end{array}$$ (3)

The total free energy has several contributions, which we now proceed to describe. The first term in Eq (3), accounts for the bending free energy, with *A*_*b*_ being the bending modulus, and Φ_*b*_ is given by [21]:

$$\begin{array}{c} \Phi_{b}=-\phi +\phi^{3}-\epsilon^{2}\nabla^{2}\phi +C_{0}\epsilon \left(1-\phi^{2}\right) \end{array}$$ (4)

where *ϵ* is the width of the interface, and *C*_0_ is the spontaneous curvature term that describes the natural trend of the vesicle to acquire a shape with a certain non-zero spontaneous curvature. In the absence of spontaneous curvature, *C*_0_ = 0, the remaining terms in Φ_*b*_ account for the mean curvature of the membrane [19, 20]. In the absence of the curvature-inducing protein, the vesicle has a curvature determined by the initial condition which has the shape of a rod-like vesicle, and *C*_0_ = 0. In a Helfrich model, the changes in (local) curvature are naturally introduced via a spontaneous curvature contribution to the energy [21]. In the present model, the spontaneous curvature is both generated and locally modified by the local concentration of the protein, whose evolution is described by Eq (2), so that the spontaneous curvature is not intrinsic but produced by the presence of the protein. Following the approach of Picallo et al. [18], we consider the spontaneous curvature term to be a function of *u*: *C*_0_ = *C*_0_(*u*) = *βu*^2^, where *β* measures the strength of the interaction, that is the ability of the protein to modify the spontaneous curvature. *A*_*b*_ is the bending modulus, which will be larger as the rigidity of the membrane grows [8]. The effects of polymerisation of the curvature-inducing protein [17] have been partially taken into account by considering a non-linear (quadratic) dependence of the spontaneous curvature *C*_0_(*u*), which considers that at least dimers of curvature-inducing protein (such as FtsZ) must have been formed in order to induce variations in spontaneous curvature.

The terms in Eq (3) corresponding to *V*_*s*_, *V*_*f*_, and *V*_*p*_ are associated with the interaction between the membrane and the protein:

$$V_{s}={\left(\phi^{2}-1\right)}^{2}{\left(u-u_{min}\right)}^{2}{\left(u-u_{max}\right)}^{2}+\lambda {\left|\nabla u\right|}^{2}$$ (5)

$$V_{f}=\phi^{2}{\left(u-u_{far}\right)}^{2}$$ (6)

$$V_{p}=\nabla \phi \cdot \nabla u$$ (7)

*V*_*s*_ and *V*_*f*_ are free energy terms that seek to concentrate the protein on the membrane. *V*_*f*_ penalises the presence of protein in the bulk away from the membrane (recall that the membrane is defined as the locus of points such that *ϕ* = 0). *V*_*s*_ contributes only when the protein is on the membrane and impedes the local concentration of protein to blow up. The term (*ϕ*^2^ − 1)^2^ hinders the diffusion of the protein away from the membrane, defined as the locus of points such that *ϕ* = 1, as it forces the free energy of the protein to achieve a minimum when the protein is located on the membrane. Furthermore, via the term proportional to |∇*u*|^2^, it allows the protein concentration to diffuse on the membrane while minimising the area of the boundary between protein-rich regions (i.e. rings) and protein-free regions. Eventually, this term produces that only one ring remains (coarsening). Describing diffusion of the protein on the membrane by means of a Laplace-Beltrami operator is not necessary within the context of a phase-field model. The fields *ϕ* and *u* are defined in the whole simulation volume. It is through the presence of specific terms in the free energy (as discussed above), that the concentration of protein is confined to the (evolving) membrane shape. Within this context, it is therefore unnecessary to resort to the Laplace-Beltrami operator to account for changes in the geometry of membrane. The parameter λ is the surface tension of the protein field and *u*_*far*_ represents the average value of protein concentration in the environment of the system and can also be taken to be null. *A*_*s*_ and *A*_*f*_ measure the strength of the different energetic contributions (interface and bulk) to the binding of the protein to the membrane. Furthermore, driven by the term proportional to |∇*u*|^2^, the protein diffuses along the axial direction to concentrate on the centre of the rod-like membrane. The concentration of protein remains uniform along the radial direction. The effects of the anisotropic spontaneous curvature created by the protein are taken into account in our model through the dependence of the spontaneous curvature of the membrane on the local concentration of protein.

*V*_*p*_ corresponds to the contribution to the free energy that allows for the membrane to undergo pinching. The physical rationale for *V*_*p*_ is as follows. During the onset of constriction, which is driven by the accumulation of protein within a ring, *V*_*p*_ has a very small contribution, as ∇*u* is very small. As constriction proceeds forward, ∇*u* gradually grows. *V*_*p*_, which penalises the simultaneous variations of *ϕ* and *u* becomes important only at the later stages of constriction. At this point, ∇*u* has become very big and *V*_*p*_ dominates over the bending energy, so that the total energy is minimised by pinching the membrane thus eliminating the bottleneck region where *V*_*p*_ is very big. The parameter *A*_*p*_ accounts in an effective manner for the presence of the proteins needed to pinch the membrane [24]. When *A*_*p*_ = 0 such bottleneck configuration is stable. On the contrary, when *A*_*p*_ ≠ 0, *V*_*p*_ is such that these bottleneck configurations are energetically unfavourable as compared to the energy corresponding to two separated vesicles. Our model does not account for the energetic contributions of the Gaussian curvature, which would be necessary for a detailed analysis of the pinching process. However, our analysis concerns the shapes of the membrane prior to pinching under different conditions regarding area conservation (or lack of it). Such shapes do not depend on the Gaussian curvature since no topological changes occur at this stage (as stated by the Gauss-Bonnet Theorem).

The last term in Eq (3), in particular the role of the parameter *σ*, is crucial in our study of the different shapes produced by the dynamics of minimisation of the free energy, *F*. The dynamic Eqs (1) and (2) can be solved under two different constraints. We may impose a restriction on the vesicle area (see [21]) that mimics the situation where a constant number of lipids forms the membrane. This leads to an interpretation of *σ* as a Lagrange multiplier that is calculated by imposing surface area conservation of the membrane [25]. We may also consider that *σ* has a given value, in which case it should be interpreted as the surface tension, which accounts for the energy associated to changes in area.

Picallo et al. [18] have analysed a simpler version of the current model which did not incorporate the *V*_*p*_ contribution to the free energy. In particular, in reference [18] a linear stability analysis was performed which allowed us to establish sufficient conditions for the onset of constriction. Such sufficient conditions were formulated in terms of the instability of small, periodic perturbations to a flat membrane with a FtsZ protein uniformly distributed over such flat surface.

Our phase-field model given by Eqs (1)–(3) is intrinsically non-linear, due to the coupling between the protein concentration and the order parameter, and the *V*_*p*_ contribution to the total free energy. Therefore, our model describes the dynamics of the membrane in an accurate way in all the regimes of its dynamics, which allows us to study the long-time behaviour of the system including large membrane deformations leading to pinching. Once such instability appears, the non-linearities of the system take over and develop the different shapes studied below. In Table 1, we report the physiological values of some of the main parameters in our model and their “phase-field” equivalencies.

**Table 1:** Table showing the relation between the main dimensionless parameters used in our simulations and their estimations in SI units available in the literature (and compiled in [22]).

Parameter	Description	Value in “PFM units”	Value in SI units
*L*	System length	24 *U*_*L*_	6 *μ*m
*A*_*b*_	Bending modulus	0.2 *U*_*E*_	0.5 pN*μ*m
*A*_*p*_	Pinching characteristic energy	0.15 *U*_*E*_	0.37 pN*μ*m
*σ*	Surface tension	10 *U*_*σ*_	50 pN/*μ*m
*β*	Intrinsic curvature of the ring	0.1 $U_{L}^{-1}$	0.4 1/*μ*m

## Dynamics of shapes

We integrate numerically Eqs (1) and (2) with periodic boundary conditions using second-order finite differences for the spatial dependence and an Euler scheme for the time dependence. Since the standard second-order finite differences is a consistent finite difference method, the time step was chosen following the Courant-Friedrichs-Lewy stability criterion: Δ*t* ≤ *c*Δ*x*, where Δ*x* is the mesh size and *c* is a positive constant [20]. We choose our units so that Δ*x* = 1. The initial conditions for the vesicle are a cylindrical shape with semi-spherical caps for the membrane. Simulations are done in 2D with axial symmetry using cylindrical coordinates. In contrast to previous (linear) models that considered the spontaneous curvature to be a constant, our model is nonlinear in the sense that the dynamics of the membrane is coupled to that of the protein through the dependence of the spontaneous curvature on *u*, which allows our model to describe the whole temporal evolution of the system from constriction to pinching. The time scale of the simulations has been set up so that pinching occurs in a time scale of the order of 20 minutes. This time scale is compatible with experimental data [4]. We consider two different situations.

### Dynamics of shapes with area conservation

**Fig 1:**
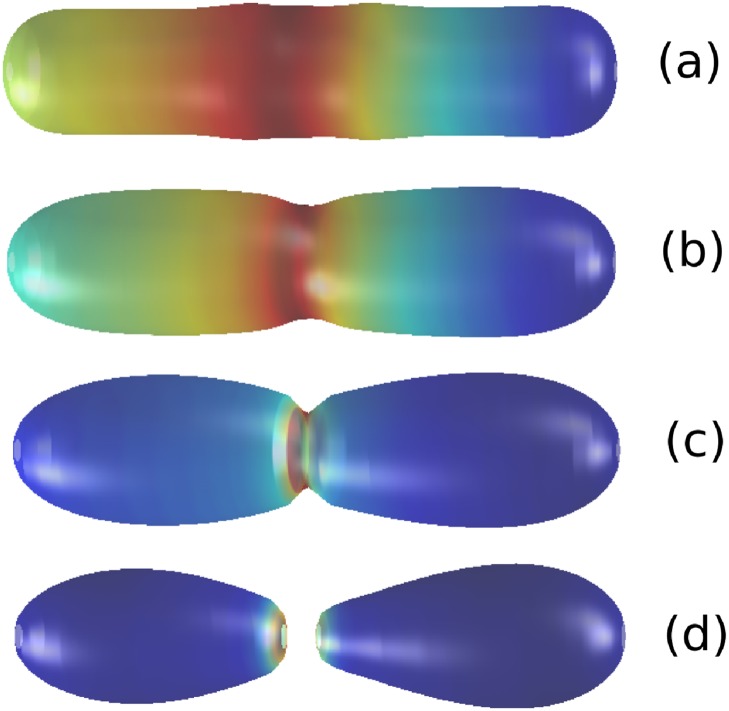
Dynamics of the simulated liposome membrane under area conservation conditions, showing the whole process from the onset of constriction (plots (a) and (b)) to eventual pinching and division (plots (c) and (d)) leading to L-form formation. Parameter values: *D*_*ϕ*_ = 1, *D*_*u*_ = 2.7, *A*_*b*_ = 0.2, *A*_*s*_ = 2, *A*_*f*_ = 2, *ϵ* = 0.01, *β* = 0.1, *u*_*min*_ = 0, *u*_*max*_ = 1, λ = 0.45, *u*_*far*_ = 0, and *u*_*h*_ = *u*_*max*_/2, *A*_*p*_ = 0.15. The colour code represents the concentration of protein on the membrane: Red corresponds to its maximum value, blue to its minimum. Regarding plot (b), we point out that since the initial conditions for our simulations exhibit cylindrical symmetry, for systems that are long enough, there is invariance with respect to the axial coordinate, which implies that the initial constriction need not be located at the exact centre of the vesicle. Plot (a), (b), (c), and (d) correspond to times *t* = 6 (0.3 mins.), *t* = 113 (5.9 mins.), *t* = 255 (13.4 mins.), and *t* = 277 (14.5 mins.), respectively.

First, we look at the shapes generated under the constraint of area conservation, which are shown in Fig 1. We have used an inhomegeneous initial condition for the concentration of protein in order to test the robustness of the steady state. In this figure we observe that, as time progresses, the protein bound to the membrane drives the onset of constriction as shown in Fig 1(a)). Consistently with the results of [8], we observe two membrane overshoots, which are the result of the resistance of the membrane to bend. At longer times (Fig 1(b) and 1(c)), a non-linear regime ensues where the influence of *V*_*p*_ (see Eq (5)) becomes dominant giving rise to pinching of the membrane as shown in Fig 1(d).

Between the onset of constriction and eventual pinching, we observe that the dynamics of the shape under constant area conditions produces the so-called L forms in agreement with the experimental observation of [4, 26] (see Fig 1(c) and 1(d)).

### Dynamics of shapes with constant surface tension

**Fig 2:**
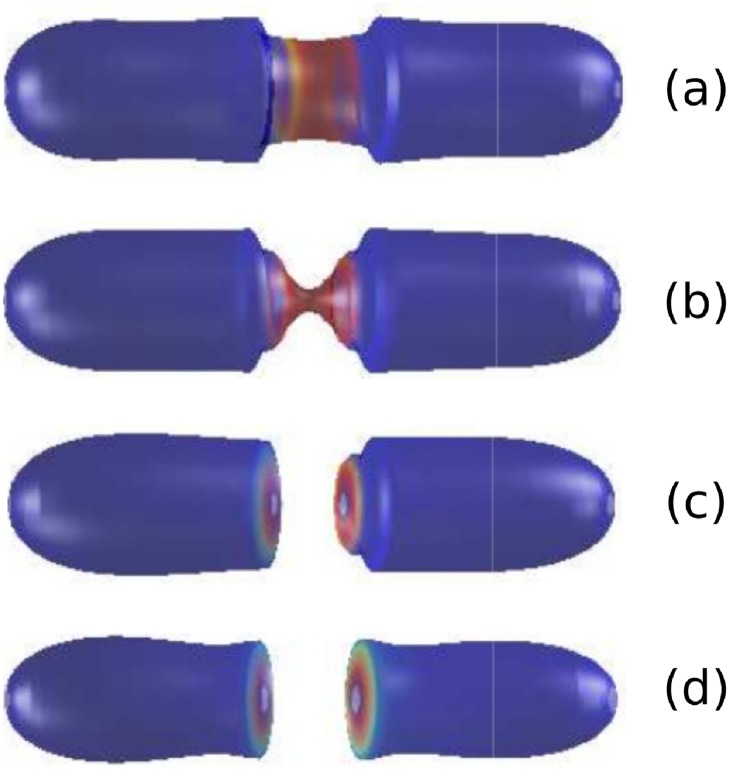
Dynamics of the membrane a rod-like vesicle with no area conservation showing the whole process from the onset of constriction (plots (a) and (b)) to eventual pinching and division (plots (c) and (d)) leading to septum formation. Parameter values: *σ* = 1. Remaining parameter values and colour code as given in Fig 1. Plots (a), (b), (c), and (d) correspond to times *t* = 122 (6 mins.), *t* = 297 (15.6 mins.), *t* = 380 (20 mins.), and *t* = 438 (23 mins.), respectively.

We now consider the behaviour of our model when the condition of area conservation has been relaxed. This is achieved by considering *σ* to have a constant value equal to the surface tension of the membrane. Results in Fig 2 show that the relaxation of such constraint leads to a completely different dynamics of the shape of the membrane. Specifically, a septum-like structure emerges spontaneously from the dynamics once we remove the constraint of area conservation (see Fig 2(b), 2(c) and 2(d)). If the system were allowed to continue to evolve according to the dynamics prescribed by our model, each of the fragments in Fig 2(d) would evolve to acquire a steady state shape similar to that of a rod-like vesicle. We have considered that the protein is initially homogeneously distributed with small random fluctuations around the mean value *u*_*h*_.

**Fig 3:**
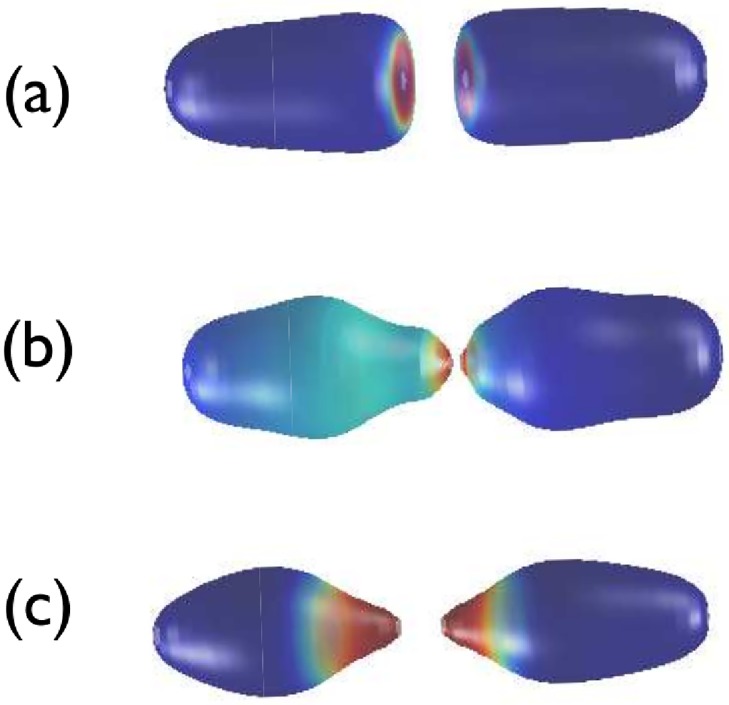
Dependence of the shape of the membrane as a function of the surface tension. Parameter values: *A*_*p*_ = 0.15. Other parameter values as given in the caption of Fig 1. The surface tension, *σ*, is: (a) *σ* = 1, (b) *σ* = 6, (c) *σ* = 15. Plots (a), (b), and (c) correspond to times *t* = 815 (42.9 mins.), *t* = 247 (13 mins.), and *t* = 100 (5.3 mins.), respectively.

The particular shape is determined by the rate at which membrane area is created. Fig 3 shows the numerical integration of our model equations under no area conservation conditions for different values of the surface tension parameter, *σ*. We show for low values of the surface tension our model gives rise to septum formation (as in Fig 2). As the surface tension increases, the pinching pattern we observe tends towards an L-form shape. When the surface tension is very high, even if the model allows for increase of area, the energy associated with such behaviour is so big that the system effectively behaves as if the constant area constraint were in place, and thus the L shapes are recovered. For conditions of strict area conservation (i.e. infinite surface tension), the pinching pattern corresponds to an L-form as shown in Fig 1.

## Discussion & conclusions

Our model allows us to describe two different scenarios, summarised in Figs 1 and 2, which correspond to two different shapes. The main difference between these scenarios is the formation of a septum (Fig 2) or lack thereof (Fig 1). Septum formation requires relaxation of the area conservation constraint. Otherwise, imposing membrane area conservation, the system divides according to an L-form-like shape.

Both L-forms and septum formation have been observed in experiments by Osawa and Erickson [4] where liposomes are under the action of constriction-inducing proteins (e.g. FtsZ) and scission-inducing proteins (e.g. FtsZ and FtsA). Our model helps to shed some light as to the conditions in which each of these scenarios are more likely to occur.

The different shapes are associated with different constraints imposed upon the dynamics of the system regarding area conservation. If the conservation of area is enforced we obtain L forms, in agreement with experimental results [4]. If, on the contrary, area is allowed to increase, the dynamics of our model produces septum formation [4]. We want to emphasise that the same model, based on a bending energy functional, allows to generate both patterns. In the case in which area is not conserved, we have a parameter, the surface tension, which accounts for the necessary energy to increase area. We have shown that by increasing the value of the surface tension, the shapes produced by the model without area conservation converge towards those obtained when area is conserved, i.e. we move from septum formation to L-form-like shapes. It is noteworthy that the characteristic time scale of the relaxation towards a L-form shape is shorter than that associated to septum formation (see Fig 3), since the energetic cost associated to the formation of a L-form pattern is lower than the septum shape.

The dispersion relation resulting from the linear analysis, thoroughly studied in our previous work [18], allows us to predict under which conditions onset of constriction occurs. Linear instability is a sufficient condition for initiation the process which eventually produces the aforementioned membrane shapes. Beyond the predictions of the linear stability analysis [18], we observe, that once the instability has given rise to formation of Z-rings the term in the free energy *V*_*p*_, which depends on the gradient of the concentration of protein, allows pinching to occur.

The purpose of the analysis presented in this paper is the study of the long-time dynamics of membrane shapes before eventual pinching. These events are prior to a change of the membrane topology and, therefore, Gaussian curvature needs not be considered during these stages. Gaussian curvature has been introduced within the phase-field formalism by Campelo [27], and, therefore, can be included in our model. Although Gaussian curvature is not going to change the qualitative behaviour of our current model, it could be of interest for a detailed characterisation of the pinching process.

## Supporting information

S1 Fig Matlab code. This file contains the matlab code used to produce the simulation results in this paper.
(PDF)
