# Supplementary material for: The dynamics of shapes of vesicle membranes with time dependent spontaneous curvature
Source: PLoS One. 2020 Jan 14;15(1):e0227562. doi: 10.1371/journal.pone.0227562 (PMC6959615; doi:10.1371/journal.pone.0227562)
Supplement: S1 Fig — This file contains the matlab code used to produce the simulation results in this paper. (PDF) [file pone.0227562.s001.pdf]

Program to calculate the dynamics of constriction

```
%%%%%%% parameters are as in Fig.2 without area conservation %%%%%%%
clear all
Nx=92;
Ny=14;
R=Ny-8;
load formerdata % load initial phi and u from a separate calculation
 with ring in the middle
et=1;
dx=1;
NF=400;
sig=0*(1:NF);
ep1=.01;
ep=ep1^2;
er1=sig;
er2=sig;
sifiu=.15;
sigma=1;
duu=.1;
Du=2.7;
D=4.5;
bet=.1;
%%%%%%%%%%%%%%%%%%%%%%%%%%%%%%%%
Aout=2;
Av=2; Afi=.2;
%%%%%%%%%%%%%%%%%%%%%%%%%%%%%%%%

%%%%%%%%%%%%%%%%%%%%%%%%%%%%%%%%%coordinates of the poles
u1=1;
u2=-.0;
u0=.5;
uout=0.0;

%%%%%%%%%%%%%%%%%%%%%%%%%%%%%%%%%%%%%%%%%%%%%%%%%%

%%%%%%%%%%%%%%%%%%%%%%%%%%%%%%%%%%%%%%%%%%%%%
No=Nx/2;
Ni=Nx/2+1;
arg=Nx:-1:Ni;
%%%%%%%%%%%%%%%%%%%%%%%%%%%%%%%%%%%%%%%%%%%%%
ra=.5:Nx-.5;
for i=1:Nx
    for j=1:Ny
        rr(i,j)=abs(ra(1,i)); % this is to calculate terms in
 cylindrical coordinates
    end
end

iter=1;
```

```
grafs          % plot phi
step=5000;     % number of time steps to plot NfXstep total iterations
dt=1e-4        % time step
%load May17s43    % uncomment in case a former saved calculation is
 needed
cont=iter;

%%%%%%%%%%%%%%% Dynamical loop %%%%%%%%%%%%%%%%%%%%%%%%%%%
for iter=cont:NF

    for iiter=1:step

        H=fi;
        lap0
        lapfi=lapH;
        H=fi;
        grad0p;
        lapfi=lapfi+g0H./rr;

        r1=(u-u1).^2;
        r2=(u-u2).^2;

        B=r1.*r2;
        rout=(u-uout).^2;


        mu=((fi-ep1.*(bet*u.^2)).*((fi).^2-1)-ep*lapfi);

        H=mu;
        lap0
        lapmu=lapH;
        H=mu;
        grad0p
        lapmu=lapmu+g0H./rr;

        F=Afi*((3*fi.^2-1-2*ep1*fi.*(bet*u)).*mu-ep*lapmu);
        F=F+rout*Aout.*(fi);

        F=F+2*Av*fi.*(fi.^2-1).*B;



        H=F;
        lap0
        lapF=lapH;
        H=F;
        grad0p
        lapF=lapF+g0H./rr;

        H=u;
        lap0
        lapu=lapH;
        H=u;
        grad0p
```

```
lapu=lapu+g0H./rr;

Fs=-sigma*lapfi;


H=Fs;
lap0
lapFs=lapH;
H=Fs;
grad0p
lapFs=lapFs+g0H./rr;

Gu=Av*(fi.^2-1).^2.*((u-u1).*r2+(u-u2).*r1);
Gu=Gu-Afi*ep1*bet*u.*mu.*((fi).^2-1);
Gu=Gu+Aout*(fi).^2.*(u-uout)-sifiu*lapfi;



Fu=-Av*D*(duu*lapu)+Gu;


H=Fu;
lap0
lapFu=lapH;
H=Fu;
grad0p
lapFu=lapFu+g0H./rr;

Ft=-sifiu*lapu;

H=Ft;
lap0
lapFt=lapH;
H=Ft;
grad0p
lapFt=lapFt+g0H./rr;
lapF=lapF+lapFt;

     %  Uncomment this seccion for area conservation
       H=lapF;
       grad0circ
       derilapF=g0iH;
       derjlapF=g0jH;

       H=lapFs;
       grad0circ
       derilapFs=g0iH;
       derjlapFs=g0jH;

       H=fi;
       grad0circ
       derifi=g0iH;
       derjfi=g0jH;

       I=sum(sum(derifi.*derilapF.*rr+derjfi.*derjlapF.*rr));
```

```
                Is=sum(sum(derifi.*derilapFs.*rr+derjfi.*derjlapFs.*rr));

                 sigma=-I/Is;

            %%%%%%%%%%%%%%%%% dynamical equations %%%%%%%%%%%%%%%%%%%%%%
            fi=fi+dt*(lapF+lapFs+lapFt);
            u=u+Du*dt*lapFu;

        end

        sig(iter)=sigma;

        h=isnan(u(Nx/2,Ny/2));
        if h==1;
            break
        end
        sigma

        %%%%%%%%%%% Figures very step iterations %%%%%%%%%%%%%%
        figure(1)

        plot(ci,(1:length(ci)),'r')
        pause(.01)
        line([sum(ci)/length(ci) sum(ci)/length(ci) ],[0 length(ci)])
        axis([0 1.2 0 length(ci)])
        grid
        hold off;

        iter

        figure(2)
        surf(u);
        shading interp
        pause(.01)

        figure(3)
        plot(sig)

        figure(4)
        grafs
        colormap jet
        pause(.01)
        Fm(:,:,iter)=fi(:,:);
        U(:,:,iter)=u(:,:);

        mm(iter)=getframe(gcf); % to make a movie

    end

    %%%%%% Movie maker %%%%%%%%%%%%
    v=VideoWriter('rename.avi');
    open(v)
    writeVideo(v,mm)
    close(v)
```

Subroutines

```
%%%%%%%% subroutine grafs.m is to plot in cylindrical coordinates %%%
%%%%%
clear XX YY ZZ ci c hi ci
nivel=-0.0;
figure(5)
[A h]=contour(fi,[nivel nivel],'k');
hold on
[A0 h0]=contour(fiini,[nivel nivel],'r');
hold off
axis equal
n=size(h);
[s t]=find(A==nivel);
[q r]=size(A);
Y1=A(1,2:r);
X1=A(2,2:r);
[xx yy]=meshgrid(1:Nx,1:Ny);
[hi,ri,ci]=griddata(xx,yy,u',X1,Y1);
i=0;
[q r]=size(ri);
for j=1:r

    for tet=0:pi/50:2*pi
        i=i+1;

        XX(i,j)=(ri(j))*cos(tet);
        YY(i,j)=(ri(j))*sin(tet);
        ZZ(i,j)=hi(j);
        c(i,j)=ci(j);
    end
    i=0;
end
figure(4)
clf
hold on

p=surf(XX,YY,ZZ,c);
set(p,'FaceColor','interp','EdgeColor','none','Facealpha',0.5)
set(gca,'Projection','perspective')
axis off
view(10,15)
camlight(20,50,'infinite')
lightangle(70,-20)
lighting gouraud
colorbar
hold off
axis image
axis vis3d
axis equal

%%%%%%%%%%%%%%%%%%%%%%%%%%%%%%%%%%%%%%%%%%%%%%%%%%%%%%%%%%%%%%%%%%%%%%
```

```
%%%%%%%%%%% Subroutine lap0.m to calculate laplacian %%%%%%%%%%%%%%%
lapH(2:Nx-1,2:Ny-1)=H(1:Nx-2,2:Ny-1)+H(3:Nx,2:Ny-1)+H(2:Nx-1,1:Ny-2)...
    +H(2:Nx-1,3:Ny)-4*H(2:Nx-1,2:Ny-1);
lapH(1,2:Ny-1)=H(2,2:Ny-1)-H(1,2:Ny-1)+H(1,3:Ny)-
H(1,2:Ny-1)+H(1,1:Ny-2)-H(1,2:Ny-1);
lapH(2:Nx-1,1)=H(3:Nx,1)-H(2:Nx-1,1)+H(1:Nx-2,1)-
H(2:Nx-1,1)+H(2:Nx-1,2)-H(2:Nx-1,1);
lapH(Nx,2:Ny-1)=H(Nx-1,2:Ny-1)-H(Nx,2:Ny-1)+H(Nx,3:Ny)-
H(Nx,2:Ny-1)+H(Nx,1:Ny-2)-H(Nx,2:Ny-1);
lapH(2:Nx-1,Ny)=H(2:Nx-1,Ny-1)-H(2:Nx-1,Ny)+H(3:Nx,Ny)-
H(2:Nx-1,Ny)+H(1:Nx-2,Ny)-H(2:Nx-1,Ny);
lapH(1,1)=H(2,1)-H(1,1)+H(1,2)-H(1,1);
lapH(1,Ny)=H(2,Ny)-H(1,Ny)+H(1,Ny-1)-H(1,Ny);
lapH(Nx,1)=H(Nx-1,1)-H(Nx,1)+H(Nx,2)-H(Nx,1);
lapH(Nx,Ny)=H(Nx-1,Ny)-H(Nx,Ny)+H(Nx,Ny-1)-H(Nx,Ny);

%%%%%%%%%%%%%%%%%%%%%%%%%%%%%%%%%%%%%%%%%%%%%%%%%%%%%%%%%%%%%%%%%%%%%%
%%%%

%%%%%%%%%%%% Subroutine gra0p.m to calculate gradient %%%%%%%%%%%%%%
%%%%
g0H(2:Nx-1,2:Ny-1)=(H(3:Nx,2:Ny-1))-H(2:Nx-1,2:Ny-1);
g0H(1,2:Ny-1)=(H(2,2:Ny-1)-H(1,2:Ny-1));
g0H(Nx,2:Ny-1)=(H(Nx-1,2:Ny-1)-H(Nx,2:Ny-1));
g0H(2:Nx-1,Ny)=(H(3:Nx,Ny)-H(2:Nx-1,Ny));
g0H(1,1)=(H(2,1)-H(1,1));
g0H(1,Ny)=(H(2,Ny)-H(1,Ny));
g0H(Nx,1)=(H(Nx-1,1)-H(Nx,1));
g0H(Nx,Ny)=(H(Nx-1,Ny)-H(Nx,Ny));
g0H(Nx-1,:)=0;
g0H(Nx,:)=0;
g0H(1,:)=0;
g0H(2,:)=0;

%%%%%%%%%%%%%%%%%%%%%%%%%%%%%%%%%%%%%%%%%%%%%%%%%%%%%%%%%%%%%%%%%%%%%%
%%%%%

%%%%%%% Soubroutine gra0circ.m to calculate Lagrange multoplier %%%%%%

g0iH(2:Nx-1,2:Ny-1)=.5*(H(1:Nx-2,2:Ny-1))-H(3:Nx,2:Ny-1);
g0jH(2:Nx-1,2:Ny-1)=.5*(H(2:Nx-1,1:Ny-2)-H(2:Nx-1,3:Ny));
g0iH(1,2:Ny-1)=.5*(H(2,2:Ny-1)-H(1,2:Ny-1));
g0jH(1,2:Ny-1)=.5*(H(1,3:Ny)-H(1,2:Ny-1));
g0iH(Nx,2:Ny-1)=-.5*(H(Nx-1,2:Ny-1)-H(Nx,2:Ny-1));
g0jH(Nx,2:Ny-1)=.5*(H(Nx,3:Ny)-H(Nx,2:Ny-1));
g0iH(2:Nx-1,Ny)=.5*(H(3:Nx,Ny)-H(2:Nx-1,Ny));
g0jH(2:Nx-1,Ny)=-.5*(H(2:Nx-1,Ny-1)-H(2:Nx-1,Ny));
g0iH(1,1)=.5*(H(2,1)-H(1,1));
g0jH(1,1)=.5*(H(1,2)-H(1,1));
g0iH(1,Ny)=.5*(H(2,Ny)-H(1,Ny));
g0jH(1,Ny)=-.5*(H(1,Ny-1)-H(1,Ny));
g0iH(Nx,1)=-.5*(H(Nx-1,1)-H(Nx,1));
g0jH(Nx,1)=.5*(H(Nx,2)-H(Nx,1));
g0iH(Nx,Ny)=-.5*(H(Nx-1,Ny)-H(Nx,Ny));
```

```
g0jH(Nx,Ny)=-.5*(H(Nx,Ny-1)-H(Nx,Ny));

g0iH(Nx,:)=g0iH(Nx-1,:);
g0iH(1,:)=g0iH(2,:);

g0jH(Nx,:)=g0jH(Nx-1,:);
g0jH(1,:)=g0jH(2,:);

g0jH(:,Ny)=g0jH(:,Ny-1);
g0jH(:,1)=g0jH(:,2);

g0iH(:,Ny)=g0iH(:,Ny-1);
g0iH(:,1)=g0iH(:,2);

 %%%%%%%%%%%%%%%%%%%%%%%%%%%%%%%%%%%%%%%%%%%%%%%%%%%%%%%%%%%%
```

Published with MATLAB® R2019b
